# Product safety aspects of plant molecular farming

**DOI:** 10.3389/fbioe.2023.1238917

**Published:** 2023-08-08

**Authors:** J. F. Buyel

**Affiliations:** Department of Biotechnology (DBT), Institute of Bioprocess Science and Engineering (IBSE), University of Natural Resources and Life Sciences (BOKU), Vienna, Austria

**Keywords:** endotoxins, expression strategy, host selection, production process, toxic metabolites, virus removal

## Abstract

Plant molecular farming (PMF) has been promoted since the 1990s as a rapid, cost-effective and (most of all) safe alternative to the cultivation of bacteria or animal cells for the production of biopharmaceutical proteins. Numerous plant species have been investigated for the production of a broad range of protein-based drug candidates. The inherent safety of these products is frequently highlighted as an advantage of PMF because plant viruses do not replicate in humans and vice versa. However, a more nuanced analysis of this principle is required when considering other pathogens because toxic compounds pose a risk even in the absence of replication. Similarly, it is necessary to assess the risks associated with the host system (e.g., the presence of toxic secondary metabolites) and the production approach (e.g., transient expression based on bacterial infiltration substantially increases the endotoxin load). This review considers the most relevant host systems in terms of their toxicity profile, including the presence of secondary metabolites, and the risks arising from the persistence of these substances after downstream processing and product purification. Similarly, we discuss a range of plant pathogens and disease vectors that can influence product safety, for example, due to the release of toxins. The ability of downstream unit operations to remove contaminants and process-related toxic impurities such as endotoxins is also addressed. This overview of plant-based production, focusing on product safety aspects, provides recommendations that will allow stakeholders to choose the most appropriate strategies for process development.

## 1 Introduction

Plants and plant cells can be used to produce active pharmaceutical ingredients, including small-molecule drug candidates and recombinant proteins (Eidenberger et al., 2023). Although recombinant proteins can be produced by many different host systems, the post-translational modifications (PTMs) carried out by plants (particularly glycosylation) can result in superior product activity (Tekoah et al., 2013; Gengenbach et al., 2019), or they can be humanized using state-of-the-art genetic engineering tools (Strasser et al., 2008; Jansing et al., 2018). The same tools can be used to modify host plant species such as tobacco (*Nicotiana tabacum*) (Menary et al., 2020a), converting them into designer hosts optimized for biopharmaceutical production (Fraser et al., 2020; Buyel et al., 2021; Huang and Puchta, 2021; Uranga et al., 2021). One example of this approach is the modification of tobacco metabolism to eliminate nicotine biosynthesis (Schachtsiek and Stehle, 2019). The production strategy can be tailored to prioritize speed (transient expression) or scalability (transgenic plants) as required for specific products and market expectations (Buyel et al., 2017; Tusé et al., 2020). Once an ideal host and production strategy have been identified, downstream processing platform technologies can be selected to ensure high product purity (Buyel et al., 2015a; Ma et al., 2015), including compliance with good manufacturing practices (GMP) even when using basic facilities for cultivation, such as greenhouses (Ma et al., 2015; Ward et al., 2021). The number of dedicated virus removal steps is often lower in PMF processes compared to those based on mammalian cells because plant cells do not support the replication of human viruses (Commandeur and Twyman, 2005; Ma et al., 2015).

These principles suggest that plants and plant cells could be widely used to produce safe biopharmaceuticals in compliance with regulatory requirements and manufacturing standards (Hundleby et al., 2022). Nevertheless, only a small number of PMF products have been approved thus far, and given the diverse production platforms involved, each of them may be regarded as unique. In contrast, microbial and animal cells have been used to produce many different approved recombinant biopharmaceutical proteins (Walsh and Walsh, 2022). Therefore, it is important to identify key factors for the design of cost-efficient, scalable, sustainable and especially safe plant-based manufacturing processes for biopharmaceutical proteins, ultimately allowing the industry to adopt the technology without reservation (Menary et al., 2020b).

**FIGURE 1:**
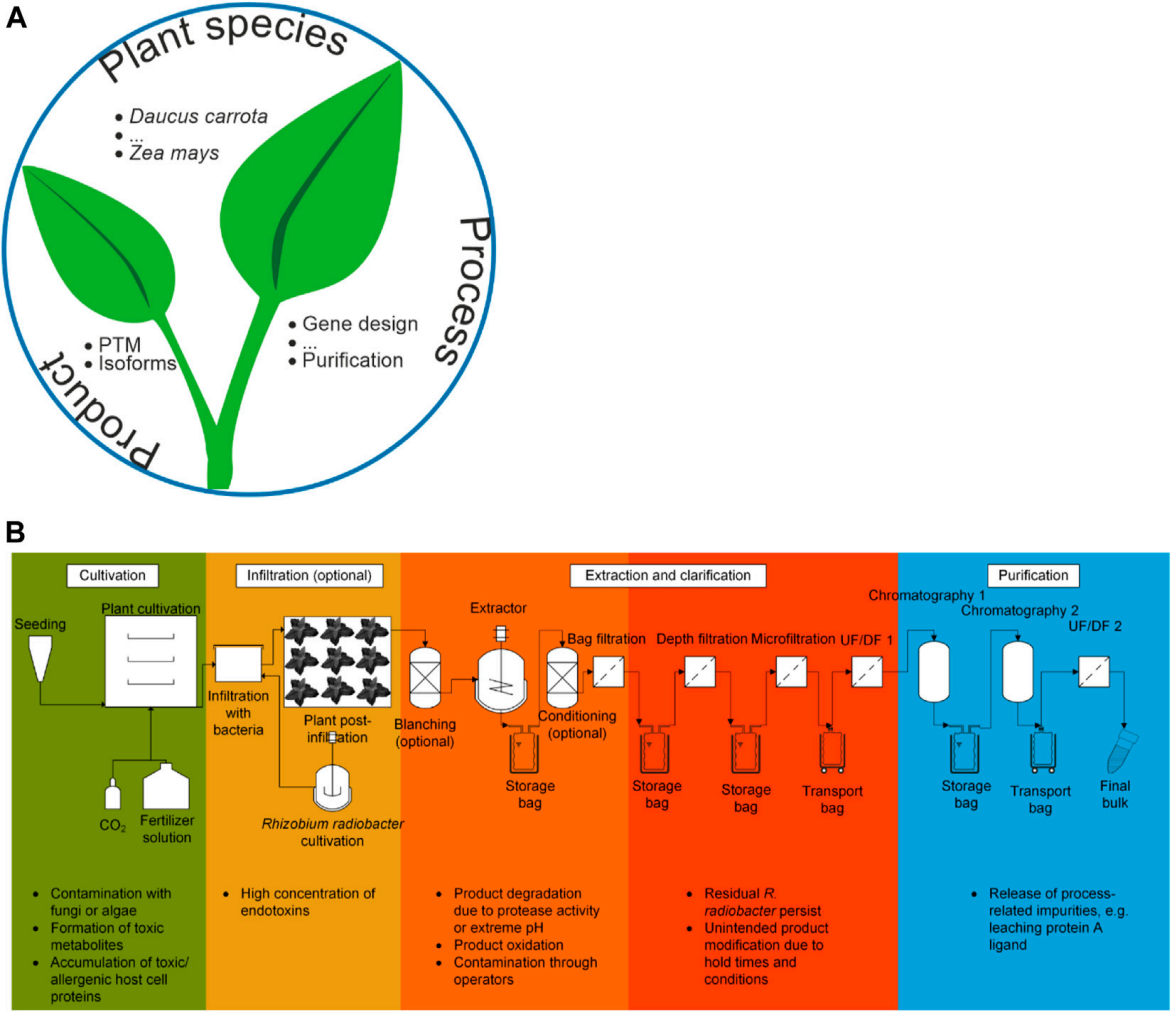
Product safety aspects of plant molecular farming. **(A)** The three major aspects that determine final product safety as discussed in this review, including some examples. **(B)** Generic process scheme for the production of recombinant proteins in plants. The cultivation is depicted as a (fully) controlled environment growth system (Section 3.3.2) but other settings can be used, such as greenhouses. The scheme can be adapted to a transgenic expression strategy by omitting the infiltration and *Rhizobium radiobacter* cultivation steps. It can also be converted to a plant cell suspension culture process by replacing the cultivation of whole plants with a bioreactor train. In the latter case, infiltration may still be relevant if plant cell packs are used for expression (Rademacher et al., 2019). Some potential risk factors are highlighted at each process step.

This review discusses the safety aspects of PMF, covering a diverse range of plant species (hosts), processes and products (Figure 1) and the associated risks (Table 1). We first consider the impact of host selection, which determines whether the presence of toxic metabolites and proteins must be taken into account. Next, we address production processes, including plant cultivation conditions, expression strategies, and purification operations. Then we turn to the product and its modification within the plant, which links back to host selection. We conclude by assessing the potential of breeding and genetic engineering to address some of the key safety concerns. This article does not consider the environmental or work-related safety of PMF (Knödler et al., 2023a), such as the release of transgenic pollen into the environment, which has been discussed elsewhere (Commandeur and Twyman, 2005). Whereas the focus of this review is biopharmaceuticals, similar considerations apply to products such as food and feed additives, albeit with differences in the mode of manufacturing and utilization. For example, food and feed additives are generally produced on a larger scale than pharmaceuticals and must remain functional after oral delivery (Barzee et al., 2022), whereas pharmaceuticals can be formulated for many different delivery modes, including oral, intravenous and intramuscular. Similarities between pharmaceutical PMF and non-pharmaceutical applications are highlighted where appropriate.

**TABLE 1:** Sources of risk in plant molecular farming that particularly affect product safety.

	Source			
Process property	Toxins	Pathogens	Oncogenes	Product modifications
Plant species	Endogenous metabolites, lectins	Toxins from algae and bacteria, attraction of disease vectors	n.a	Host-specific glycosylation
Cultivation conditions	Toxins from microbial contamination, higher metabolite levels	Contamination with microorganisms or animals	n.a	Proteolytic degradation or truncation
Expression strategy	Endotoxins from *R. radiobacter* ^a^	Residual *R. radiobacter* ^a^	Promoter sequences or coding sequences of recombinant proteins	Incomplete PTMs due to overexpression
Subcellular targeting	n.a	n.a	n.a	Incomplete processing, aberrant PTMs
Harvesting	n.a	Contamination via personnel	n.a	Oxidation or degradation due to storage
Extraction conditions	Increased metabolite solubility	No microorganism inactivation	Increased host DNA solubility and size	Oxidation
Purification strategy	Insufficient removal	Insufficient removal	Insufficient removal	Insufficient removal of inactive product isoforms or degradation products
Storage	Re-contamination	Re-contamination	n.a	Degradation, oxidation, truncation, or aggregation

^a^ *Rhizobium radiobacter* was formerly known as *Agrobacterium tumefaciens*; n.a.—not applicable; PTMs, post-translational modifications.

## 2 Host-related safety aspects of plant molecular farming

### 2.1 Host-specific harmful metabolites and proteins

#### 2.1.1 Small-molecule metabolites

Host cell components are defined as process-related impurities in all expression systems (Argentine et al., 2007; Arfi et al., 2016; Jones et al., 2021). In some cases, such molecules are directly toxic, such as the lipopolysaccharides known as endotoxins produced by Gram-negative bacteria (Section 3.1) (Serdakowski London et al., 2012). In contrast to these large cell wall components that are easily detected in specific assays, plants and plant cells also contain diverse metabolites with a wide dynamic range of concentrations, including pigments (e.g., chlorophyll) and polyphenols (Moore et al., 2014; Wang et al., 2019). The specific purpose or benefit of these complex small molecules may not readily be apparent, but they are often intrinsically bioactive (Acamovic and Brooker, 2005; Wink, 2009; Napagoda et al., 2022). Accordingly, they are exploited as food additives, cosmetic ingredients and pharmaceuticals, such as the extraction of the anti-cancer drug paclitaxel from medicinal plants (Pereira et al., 2012; Buyel, 2018) and derived cell cultures (Ochoa-Villarreal et al., 2016). However, where such bioactive compounds are present in PMF hosts used for the production of recombinant proteins, they are treated as impurities that must be removed during purification (Table 2). For example, nicotine is purified from tobacco for use as a pharmaceutical, including nicotine replacement therapy and the treatment of mild cognitive impairment (Sanchez-Ramos, 2020; Kheawfu et al., 2021), but when tobacco is used to produce recombinant monoclonal antibodies the nicotine is an unwanted impurity (Ma et al., 2015).

**TABLE 2:** Examples of toxic compounds found in the plant hosts used for PMF and associated microorganisms.

Molecule name	Molecule type	Molecular mass (Da)	Host species	Trivial name	Concentration (mg kg^−1^ fresh biomass)	Dose (mg kg^−1^ body mass)	Dose type (−)	Reference species	Route of administration	Ref
Anabasine	Alkaloid	162	*N. tabacum*	Tobacco	250	11–16	LD_50_	Mouse	i.v	Sisson and Severson (1990), Lee et al. (2006)
Ciguatoxin	Polyether	1,100–1,300	*G. toxicus*	n.a	n.a	0.0003	LD_50_	Mouse	n.a	Lewis (2000), Fusetani and Kem (2009)
Cyanogenic glycosides	Glycosides	250–900	Diverse, e.g., *Eucalyptus cladocalyx*	Sugar gum	4,000–15,000	4.3	LD_50_	Rat	i.p	Toxic Rep Ser (1993)
						15	LD_50_	Rat	p.o	
						4.9–5.9	LD_50_	Mouse	i.p	
						2.9	LD_lo_	Human	p.o	
Gluten	Storage protein	30,000–100,000	cereal crops, e.g., *T. aestivum*	Diverse, e.g., wheat	40,000–90,000	10–100 [mg per person per day]	“safe range”	Human	p.o	Hischenhuber et al. (2006), Cohen et al. (2019), Pronin et al. (2020)
Nicotine	Alkaloid	162	*N. tabacum*	Tobacco	20,000–50,000	6–13	LD_50_	Human	p.o	Mayer, 2014; Henry et al. (2019)
Saxitoxins	Complex heterocyclic compound	299	Dinoflagellates, e.g., *L. wollei*	Diverse, n.a	n.a	0.0005	NOAEL	Human	p.o	Weirich and Miller (2014)
Viscumin	Ribosome-inactivating protein	62,628	*V. album*	Mistletoe	n.a	0.002^a^	LD_50_	Mouse	i.v	Olsnes et al. (1982)

^a^ assuming ∼0.03 kg body mass per mouse; LD_50_, median lethal dose; LD_lo_, minimal lethal dose; i.m., intramuscular; i.p., intraperitoneal; i.v., intravenous; NOAEL, no observed adverse effect level; p.o., peroral; s.c., subcutaneous.

Solanaceous plants like tobacco, pepper (*Capsicum annuum*), potato (*Solanum tuberosum*) and tomato (*Solanum lycopersicum*) are attractive for PMF applications because they produce large amounts of biomass [e.g., 100,000–500,000 kg ha^−1^ a^−1^ for tobacco (Stoger et al., 2002; Huebbers and Buyel, 2021)]. However, they also contain undesirable or even toxic alkaloids like capsaicin, solanine, anabasine and nicotine (Green et al., 2013; Stephan et al., 2017; Günthardt et al., 2018). The latter has an estimated median lethal dose (LD_50_) of 6–13 mg kg^−1^ body mass in humans (peroral uptake; intravenous probably less) (Mayer, 2014), but concentrations as low as 0.025 mg kg^−1^ may trigger biological reactions such as altered leg extensor torque (Mündel et al., 2017). In the case of anabasine, the teratogenic potential rather than acute toxicity is the major concern (Keeler et al., 1984; Green et al., 2013), even though it is difficult to identify a suitable model system (Welch et al., 2014).

Similarly, cyanogenic glycosides are amino acid-derived compounds found in many plants, including crops, at various stages during their life cycle, depending on the nutrient supply (Gleadow and Møller, 2014; Lechtenberg et al., 2005-2010). This complex group of molecules has probably emerged in defense against herbivores, and their toxicity stems from the release of hydrogen cyanide upon contact with specific β-glucosidases. Depending on the plant species and tissue, cyanogenic glycosides may be present at concentrations up to ∼8 g kg^−1^ dry plant matter. For example, the concentration in young *Eucalyptus cladocalyx* leaves is twice that of old leaves (Gleadow and Woodrow, 2000). Similar concentrations are found in bamboo (*Bambusa vulgaris*, 1–8 g kg^−1^) (Nyirenda et al., 2021). Some plant compounds are even more toxic, including saponins and glycoalkaloids (Wink, 2009; Napagoda et al., 2022; Rasool et al., 2022).

It is therefore necessary to remove such metabolites during product purification, depleting them not only below the level of toxicity but below the minimum effect level, which may be unknown or difficult to determine. Furthermore, the specific compound in a plant extract that triggers a given biological reaction (such as the impairment of immune responses) may not yet be known (Harwanto et al., 2022; Urbański et al., 2023). Establishing and updating systematic databases of plant-derived toxins (Günthardt et al., 2018) can help to ascertain the risks associated with certain plant hosts in a rational manner. The corresponding quantitative assays are also necessary for the successful, targeted and rational development of safe processes.

One practical example of metabolite removal is the production of monoclonal antibodies in tobacco for human clinical testing. Nicotine was depleted below the limit of detection by applying a simple two-stage purification process consisting of capture chromatography using protein A resin and a polishing step using ceramic hydroxyapatite (Ma et al., 2015). This was possible primarily because the size (or mass) of the monoclonal antibody product and nicotine (i.e., three orders of magnitude) as well as their surface properties differ substantially (e.g., in terms of charge and hydrophobicity). Similar results have been reported by others (Fu et al., 2010). Efficient separation can be more challenging if the product is also a small molecule, especially if the physicochemical properties of the product and impurities are similar (e.g., in terms of solubility). Specifically, this would rule out the use of porous membrane-based unit operations such as ultrafiltration/diafiltration, which can remove small-molecule impurities during buffer exchange operations when purifying larger proteins (Opdensteinen et al., 2018). It is therefore useful to select host plants in which there are no known toxic metabolites or where such metabolites are easy to separate from the product (Table 3). Accordingly, several food plants or cell cultures derived from them have been used for the production of safe biopharmaceuticals, including carrot (*Daucus carota*), lettuce (*Lactuca sativa*), maize (*Zea mays*), barley (*Hordeum vulgare*) and rice (*Oryza sativa*) (Xu et al., 2011; Grabowski et al., 2014; Mirzaee et al., 2022; Ganesan et al., 2023). But even food crops can contain low concentrations of toxic alkaloids that need to be removed during processing, such as lupinin from lupin (*Lupinus mutabilis*) (Kaiser et al., 2020; Griffiths et al., 2021). Conventional breeding and genetic modification can be used to deplete or even fully remove such metabolites, as discussed in more detail later (see Section 5). Overall, the risk posed by plant-derived small-molecule impurities is low if the product is a recombinant protein because purification schemes typically include size-based fractionation steps to remove protein aggregates and degradation products, and these steps also ensure the removal of alkaloids and other bioactive metabolites.

**TABLE 3:** Overview of safety aspects of selected plant host species used in molecular farming.

				Examples of harmful agents		
Plant species	Trivial name	Frequent cultivation strategy	Frequent expression strategy	Metabolites	Proteins^a^	Pathogen-related
*Daucus carota*	Carrot	Suspension culture	Transgenic	Carotatoxin Crosby and Aharonson (1967)	*Dau c 1* Hendrich et al. (2023)	n.f
*Hordeum vulgare*	Barley	Intact plants	Transgenic	Hordenine Liu and Lovett (1993)	*Protease inhibitors, 15-kDa* Mena et al. (1992); Wróblewska et al. (2022)	Mycotoxins Drakopoulos et al. (2021)
*Lactuca sativa*	Lettuce	Intact plants	Transgenic	n.f	*EP1-like protein* Sekiya et al. (2020), *thaumatin-like protein* Muñoz-García et al. (2013), *aspartyl protease* Muñoz-García et al. (2013), *sesquiterpene lactones*, e.g., lactucin Paulsen and Andersen (2016), *Lac s 1* Hartz et al. (2007)	Mycotoxins, e.g., tentoxin and tenuazonic acid Kłapeć et al. (2021); Miranda-Apodaca et al. (2023)
*Nicotiana benthamiana*	Australian tobacco	Intact plants	Transient	Alkaloids, e.g., nicotine Hayashi et al. (2020)	No specific reports, but probably similar to tobacco	Sphinganine-analog mycotoxins Rivas-San Vicente et al. (2013)
*Nicotiana tabacum*	Tobacco	Intact plants; suspension culture	Transgenic	Alkaloids, e.g., nicotine Hayashi et al. (2020)	Allergies reported but allergen unknown, probably pollen-related Ortega et al. (1999); Bonamonte et al. (2016)	Mycotoxins el-Maghraby and Abdel-Sater (1993)
*Oryza sativa*	Rice	Intact plants; suspension culture	Transgenic	n.f	*Glyoxalase I* Usui et al. (2001), *Ory s1* Sharma et al. (2009)	Diverse, e.g., aflatoxin B1 Rofiat et al. (2015) and zearalenone Joo et al. (2019)
*Zea mays*	Maize	Intact plants	Transgenic	n.f	*Lipid transfer protein* Pastorello et al. (2000)	Fumonisins Duvick (2001)

^a^ allergens are in italics; n.f., none found.

#### 2.1.2 Plant host cell proteins

Plants not only contain toxic metabolites but also some harmful proteins. The most toxic proteins are ribosome-inactivating toxins like ricin or viscumin but the plants that produce such toxins are not used as PMF hosts (Olsnes et al., 1982; Worbs et al., 2011). However, other lectins such as rice bran agglutinin (UniProt ID Q0JF21; ∼22 kDa) or pea (*Pisum sativum*) lectin (UniProt ID P02867; ∼30 kDa) are present in PMF food crops (Miyoshi et al., 2001; Kabir et al., 2013). These proteins can arrest the cell cycle, inhibit proliferation or trigger apoptosis in animals and therefore confer a relevant safety risk that should be monitored (Jiang et al., 2015). Due to their size, they may co-purify with products such as cyanovirin-N (∼11 kDa) (Opdensteinen et al., 2018), but should be easy to separate from large proteins like antibodies (∼150 kDa) (Ma et al., 2015). However, the carbohydrate-binding activity of many lectins causes them to bind glycosylated target proteins, which can result in co-purification. Similar nonspecific interactions have been reported between Chinese hamster ovary (CHO) host cell proteins (HCPs) and monoclonal antibodies (Li, 2022). Conditions that suppress such interactions should be identified during downstream process development.

The presence of glutens is another protein-based risk, which is particularly relevant when using cereal crops as PMF hosts (Ito, 2015; Abedi and Pourmohammadi, 2020). Glutens are diverse proteins that can be classified as glutenins or gliadins (also known as Osborne fractions) (Osborne, 1907; Biesiekierski, 2017). These proteins are not toxic *per se*, but they are present at much higher concentrations than most toxic proteins and are potent allergens. In wheat (*Triticum aestivum*), 40–90 g of gluten is present per kilogram of wheat flour (Pronin et al., 2020). Glutens can trigger immune responses at concentrations of ∼12 mg kg^−1^ body mass in humans (Lähdeaho et al., 2011; Cabanillas, 2020; Taraghikhah et al., 2020). Whereas some tolerance may be built up in celiac disease patients (Elli et al., 2020), a safety threshold of 10–100 mg per person per day has been proposed (Hischenhuber et al., 2006; Cohen et al., 2019). Importantly, glutens are soluble in water and are stored in the seeds, where recombinant proteins tend to be targeted in cereals because this enhances product stability (Tosi et al., 2011; Arcalis et al., 2014). The concentration of these allergens in primary seed extracts is therefore high. Glutens are thermostable (Biesiekierski, 2017) and range in molecular mass from ∼30 to >100 kDa (Tosi et al., 2011), so they can be difficult to separate from target proteins by blanching/heating (Buyel et al., 2016) or ultrafiltration/diafiltration (Opdensteinen et al., 2018). Although the presence of gluten is challenging in terms of downstream process development, the overall safety impact is low. Specifically, glutens are easy to detect (Schubert-Ullrich et al., 2009) and pharmaceutical proteins must exceed 95% purity (Jin et al., 2018). In the unlikely event that a PMF product contains 5% gluten, and large doses of the product are required (e.g., 0.05 g anti-Ebola antibody per kilogram of body mass every 3 days (Davey et al., 2016)), a 70-kg patient would be exposed to an average of ∼175 mg gluten per day, which would be about twice the safe threshold. Although it is unlikely that a single compound would account for all impurities in a product, this estimate underlines the importance of removing even compounds that may be regarded moderate safety risks, such as allergens. This applies especially in cases where high doses (up to several grams per person) of product are required, as might be the case in post-exposure prophylaxis, the treatment of acute disease (Taylor et al., 2021; Hwang et al., 2022), or cancer therapy (Hendrikx et al., 2017). It is also relevant for non-antibody products that cannot be captured by affinity chromatography, and non-pharmaceutical products such as food additives where any form of chromatography would too expensive.

Importantly, the number, abundance and activity of hazardous proteins can be reduced, in some cases to below the level of detection, through process development (see Section 3.4) and genetic engineering strategies (see Section 5). For example, the majority of plant host cell proteins can be removed by anion exchange chromatography (Buyel and Fischer, 2014a; Bernau et al., 2022).

### 2.2 Contamination by disease vectors and plant pathogen products

In addition to harmful molecules produced by plants, PMF hosts may also attract pests and pathogens that can directly harm humans or produce toxic proteins and metabolites, which is an active area of research in the context of food safety (Fletcher et al., 2013; Sobiczewski and Iakimova, 2022). For example, fungi that infect cereals produce (ergot) alkaloids and carcinogenic mycotoxins (Hulvová et al., 2013; Florea et al., 2017; Sweany et al., 2022), the latter including aflatoxin B1 which is toxic at micromolar concentrations (Bianco et al., 2012; Marchese et al., 2018). Similarly, prokaryotic blue green algae (cyanoprokaryota) such as *Lyngbya wollei* and eukaryotic green algae (chlorophyta) such as dinoflagellates (e.g., *Ostreopsis siamensis* and *Gambierdiscus toxicus*) can colonize human environments (Hofbauer, 2021) such as personal aquariums, irrigation/drainage gullies or flood tables (see Section 3.3) and the corresponding fertilizer reservoirs. Algae can spread through the air, and also proliferate in soil or on the stone wool blocks often used to support plant growth in PMF. The risk to biomanufacturing reflects the ability of algae to produce allergens and toxins such as ciguatoxin and maitotoxin (both from *G. toxicus*) that cause diarrhea and vomiting in humans (Friedman et al., 2017; Hofbauer, 2021) or even death (Ohizumi and Yasumoto, 1983). Specifically, the LD_50_ of ciguatoxin in mice is ∼250 ng kg^−1^ when administered intraperitoneally (Lewis, 2000) and maitotoxin has a minimal lethal dose of ∼170 ng kg^−1^ (Bagnis et al., 1980; Ohizumi and Yasumoto, 1983). Likewise, cyanoprokaryota produce saxitoxins such as *L. wollei* toxin-1, with a no observed adverse effect level (NOAEL) of ∼500 ng kg^−1^ body mass following peroral uptake in humans (Weirich and Miller, 2014). For intravenous pharmaceuticals, the NOAEL is likely to be lower.

Some fungal (*Alternaria infectoria*) and bacterial (*Erwinia persinicus*) pathogens of plants may cause opportunistic infections in humans. For example, *Rhizobium radiobacter*, which is widely used for transient expression in PMF applications, can cause bacteremia and keratitis as recently reviewed (Kim et al., 2020). However, the number of reported cases is extremely low (<50 in the available literature) despite the ubiquitous nature of the species in soil and artificial environments such as laboratories (Dessaux and Faure, 2018; Zhu et al., 2020). Furthermore, most of the patients suffering from a sporadic disease that seemed to be related to *R. radiobacter* infection were immunocompromised or the infection site was related to surgery or the eye (Kim et al., 2020), where the adaptive immune system is particularly weak (Akpek and Gottsch, 2003). Accordingly, the risk of infection with plant pathogens appears to be minimal for humans, especially given that pharmaceutical products undergo (several) sterile filtration steps or even more stringent size-based separation (e.g., ultrafiltration/diafiltration or size-exclusion chromatography) that will remove any intact cells (Holtz et al., 2015; Ma et al., 2015). Endotoxins present a more relevant, process-related risk specifically associated with *R. radiobacter* and transient expression (see Section 3.2).

In contrast to such bacteria, plant viruses do not infect or replicate in human cells and are therefore unlikely to cause diseases. Plant viruses can be found in association with humans but the link is thought to be indirect–for example, tobacco mosaic virus RNA was found in human saliva, but its presence was attributed to smoking rather than an infection (Balique et al., 2012). Similarly, tobacco DNA was detected in ventilator-associated pneumonia patients who were smokers (Bousbia et al., 2010).

Plants may also attract insects that can be vectors of human diseases. For example, volatiles (especially terpenoids) released from certain plant species can attract mosquitos such as *Anopheles gambiae* (Nyasembe et al., 2012; Nikbakhtzadeh et al., 2014), a key malaria vector. Furthermore, the plant species on which the mosquitos feed can also affect the viability of *Plasmodium falciparum* (Hien et al., 2016), one of the parasites that causes malaria. Specifically, mosquitos feeding on fruits of *Mangifera indica* instead of cuttings from *Thevetia neriifolia* or a glucose control were ∼50% less likely to survive over 7 days and the mean number of developing oocysts in the guts of infected female mosquitos was reduced by ∼60%. Certain plant species or cultivation conditions may also attract rodents that carry pathogens.

The risk attributed to plant pathogens, insects and other animals in the context of PMF is low. For example, PMF crops such as tobacco only produce low concentrations of terpenoids that are unlikely to attract mosquitoes (Lücker et al., 2004). In moderate climate zones, such disease vectors are in any case unlikely to be present in the vicinity of a manufacturing site. In general, PMF cultivation conditions do not support many of the pathogens discussed above, and the natural microbiome of plants can reduce the fitness of pathogens such as *P. falciparum* (Bassene et al., 2020). As discussed below, many facilities and process design options exist to minimize or even exclude risks associated with pests and pathogens, including UV lamps or ozone generators to inactivate algae and bacteria in irrigation systems or carried by personnel, a controlled environment, and traps (e.g., mouse traps and yellow sticky traps for insects) to protect the cultivation area from pathogens spread by animals.

## 3 Potential risks arising from bioprocess design

The decisions made during process design can greatly affect product safety. Certain process steps are directly intended to focus on safety, including low-pH hold steps for virus inactivation (Mazzer et al., 2015), but other choices can have unintentional effects and should be avoided or mitigated.

### 3.1 Expression cassette elements

Oncogenes or parts thereof may be used as products or as building blocks for expression vectors, including regulatory elements to enhance product accumulation. As a product-related example, oncogenic protein E7 from human papillomaviruses binds to the retinoblastoma protein and is necessary to maintain the viability of papillomavirus-induced tumors, as found in the commonly-used HeLa cell line (Nishimura et al., 2006). However, the E7 protein has also been produced in plants (and many other host systems) as a vaccine candidate for the treatment of infections with human papillomavirus 16 (Venuti et al., 2009; Buyel et al., 2012). Accordingly, the vaccine product could potentially contain residual host cell DNA including sequences encoding the oncogenic recombinant protein. In the specific case of E7, the coding sequence had been mutated to render the protein non-tumorigenic and thus mitigate this risk (Smahel et al., 2001). However, such solutions require precise knowledge about the protein binding/interaction sites, which may not always be available.

The risks associated with such oncogenic DNA can also include regulatory sequences, and the likelihood that residual DNA could transform animal cells and ultimately trigger tumor development has been debated (Peden et al., 2006). Models have been built to assess the associated risk (Yang et al., 2010), which seems to be negligible based on evidence from multiple studies (Palladino et al., 1987; Dortant et al., 1997). Specifically, 1–10 g of residual host DNA was deemed necessary for tumor induction (Sheng et al., 2008), whereas the regulatory threshold for host cell genomic DNA in pharmaceutical products is ∼0.1–1.0 ng per dose (Wang et al., 2012). Given that sensitive PCR-based detection methods are available and that DNA is a highly charged polymer that can be removed efficiently by anion exchange chromatography (Stone et al., 2018), the risk associated with residual DNA is small. If necessary, an enzymatic treatment step can reduce the residual DNA burden further and thus improve product safety (Kawka et al., 2021).

### 3.2 Expression strategy and endotoxins

Endotoxins are a well-known risk factor in biomanufacturing because they are strong activators and modulators of the human immune system, leading to septic shock (Opal, 2010). Such toxins are abundant in processes where Gram-negative bacteria are used as hosts (Petsch and Anspach, 2000), but they are also relevant in PMF due to the deliberate use of bacteria for gene transfer and also the presence of adventitious bacteria on or within plant tissues. In the scalable transgenic system (Buyel et al., 2017), the endotoxin load is typically low because the bacteria used for gene transfer are killed after the transgenes are stably integrated into the plant nuclear or plastid genome (Herrera-Estrella et al., 2005). Therefore, the only Gram-negative bacteria present will be those naturally occurring on the plant surface, such as *Pseudomonas* spp. (Compant et al., 2019). In contrast, transient expression (Tusé et al., 2020) requires that plants are infiltrated with the Gram-negative bacterium *R. radiobacter* (Spiegel et al., 2019), and the stress involved in this process also stimulates endotoxin production as well as secondary metabolite synthesis in plants (Buyel et al., 2015b). Accordingly, the concentration of endotoxins can increase 200-fold to ∼10^4^ EU per milligram of total protein (Arfi et al., 2016), which is ∼3 × 10^4^ EU mL^−1^. This is in the same range as *Escherichia coli* lysate (10^3^–10^5^ EU mL^−1^) (Szermer-Olearnik and Boratyński, 2015), and is above the regulatory threshold of 5 EU kg^−1^ body mass h ^−1^ (Hirayama and Sakata, 2002), even assuming microgram doses of protein (Cummings et al., 2014). Accordingly, endotoxins have to be removed, particularly when the product is manufactured by transient expression. Dedicated methods such as phase separation (Aida and Pabst, 1990), affinity capture (Anspach, 2001), chromatography (Serdakowski London et al., 2012) and ultrafiltration/diafiltration (Jang et al., 2009) can be used for this purpose, as demonstrated for a range of plant extracts (Arfi et al., 2016). However, many of these steps are typically included in common downstream processing schemes (Ma et al., 2015; Opdensteinen et al., 2018; Knödler et al., 2023b), so additional effort is not usually required for endotoxin removal, as long as the process is monitored carefully.

A hybrid approach is the use of inducible transgene expression (Mortimer et al., 2015; Hahn-Löbmann et al., 2019). This strategy has the same development times as the transgenic approach but facilitates time-bound product expression, thereby minimizing toxic effects of the latter on plant development and growth. In terms of product safety, it is similar to transgenic plants (low endotoxin levels and absence of *R. radiobacter*) and the induction agent should be selected to ensure efficiency (active at low concentrations), easy removal (e.g., ideally a small molecule such as ethanol) and lack of toxicity.

### 3.3 Cultivation conditions

Importantly, regardless of the expression strategy and cultivation conditions, PMF products can be manufactured without animal-derived components because defined or vegan fertilizer/media can be used for the cultivation of plants, plant cells and *R. radiobacter* (Houdelet et al., 2017; Leth and McDonald, 2017; Geng et al., 2019; Kang et al., 2022). Therefore, contamination with pathogens and harmful agents such as prions in substrates can be ruled out, which increases the safety of PMF products.

#### 3.3.1 Plant cell suspension cultures

Bioreactors suitable for conventional microbial and mammalian cell cultures can also be used for plant cells [and even plant tissues and intact plants (Murthy et al., 2023)] with little or no modification (Holland et al., 2013). These reactors provide a high degree of process containment and minimize or even eliminate some of the risks discussed above (Huang and McDonald, 2012). For example, bacteria that colonize plant surfaces will not be found in a bioreactor. Nevertheless, care must be taken when inoculating and harvesting the reactors, especially when large volumes (i.e., several liters) are handled during the late stages of a typical reactor seed train, because sterility can be difficult to ensure, as is well known for other bioprocesses (Müller et al., 2022). The contamination risk can be reduced if, for example, orbitally shaken (Raven et al., 2014) or airlift/bubble reactors (Wilson and Roberts, 2012) are used because these contain fewer moving parts, grommets and fittings than stirred-tank reactors (Werner et al., 2018). Similarly, single-use reactors can reduce cross-product contamination risks (Raven et al., 2011). The use of photobioreactors also enhances safety because the autotrophic cultivation of plant cells in such reactors does not require organic carbon sources in the culture medium (Legrand et al., 2021), effectively depleting it of a substrate necessary for the growth many contaminating bacteria, yeast and fungi (although phototrophic bacteria and algae remain a contamination risk). The cultivation of plant cells in photobioreactors also requires the accumulation of chlorophylls and other pigments, and these compounds may unintentionally interact with product molecules (see Section 3.4.2). Another drawback of photobioreactors is that they typically use non-standard designs, such as tubular geometry, to ensure sufficient illumination (Chanquia et al., 2022).

Plant cells in suspension often have a tendency to adhere to even stainless-steel surfaces in a bioreactor (Holland et al., 2017). This not only limits the bioprocess operation time but may also interfere with cellular metabolism by limiting the oxygen and/or nutrient supply in the resulting cell clusters. These suboptimal conditions can lead to cell stress, autophagy (e.g., of peroxisomes) and cell death (Voitsekhovskaja et al., 2014; Tyutereva et al., 2018; Ma et al., 2022), which may cause (partial) product degradation or modification, ultimately increasing product heterogeneity and reducing activity. Also, if cells begin to decompose in these surface aggregates and then re-enter the bulk fermentation broth, the molecules they release may trigger unwanted signaling cascades in the living cells, reducing overall productivity (Salguero-Linares and Coll, 2023). Therefore, production cell line development and cultivation protocols should focus on low adhesion and low aggregation properties as well as monitoring strategies to ensure that product quality is not compromised.

#### 3.3.2 Cultivation of intact plants

Plant cultivation in the open field is currently suitable only for small-molecule pharmaceutical products like morphine, which is extracted from opium poppy straw (Krikorian and Ledbetter, 1975). These molecules have a simple structure, a well-defined conformation, and are typically isolated using organic solvents that have the added value of acting as disinfectants (Kuyukina et al., 2014). Therefore, product quality control is straightforward (e.g., LC-MS analysis) and any contaminants are effectively removed by the harsh extraction conditions.

In contrast, to date protein-based pharmaceuticals have been produced in plants grown indoors. This avoids any unpredictable effects of the variable external environment and ensures compliance with GMP requirements. Specifically, recombinant protein extraction typically relies on aqueous buffers (Buyel et al., 2015a) that do not inactivate pathogens introduced by pest insects and rodents (see Section 2.2). Additional factors that bar open field cultivation are heavy metal ions, pesticides and anthropogenic toxic pollutants that can contaminate soils (Sigmund et al., 2022; D'Angelo et al., 2013) and plant tissues, and potentially the final product (Zeng et al., 2019; Zhang et al., 2020).

Such risks can be averted if plants are cultivated in greenhouses, as reported for several GMP-compliant processes for the production of monoclonal antibodies and vaccines (Ma et al., 2015; Ward et al., 2021). Also, well-defined growth supports such as stone wool blocks can be used in this setting and can be combined with automated hydroponic irrigation systems. The closed environment facilitates effective pest control and allows strictly regulated personnel access, which minimizes the risk of contamination with pathogens. However, process control is still limited in such a setting. For example, yields of the same antibody product can fluctuate between 2 and 6 g per 200-kg batch of plants due to seasonal effects and variable weather (Sack et al., 2015). More importantly, product integrity can be compromised by protease activity, for example, under conditions of intense light or high temperature that cannot be mitigated by climate control (Knödler et al., 2019). Even if climate control maintains cultivation conditions within specifications, leaves can become hotter than the surrounding environment due to intensive insolation (Huebbers and Buyel, 2021). Intense light can also trigger the synthesis of potentially harmful metabolites (Buyel et al., 2015b; Thoma et al., 2020) (see Section 2.1) that need to be removed during downstream processing. Because greenhouses are typically non-sterile environments, it is likely that algae will start to grow on surfaces and in the fertilizer solution, especially if the tanks and gullies/flood tables are not properly covered. As discussed above, these prokaryotic and eukaryotic algae can be harmful or may secrete toxic compounds (see Section 2.2). Therefore, in-line UV light or ozone generators should be installed to reduce the impact of algae (Sharrer and Summerfelt, 2007).

Closed cultivation facilities achieve an even higher degree of process control than greenhouses. These facilities are designed to eliminate any environmental impact on plant growth by providing a complete artificial climate: temperature, humidity and irrigation as well as light and potentially gas composition (Farhangi et al., 2023). The terminology used for closed cultivation facilities can be misleading and ambiguous. For example, they are often called “vertical farms” because multiple vertically-stacked cultivation layers can improve cost-efficiency, but single-layer designs can be used as well (Huebbers and Buyel, 2021). The alternative term “indoor farm” or “indoor agriculture” is also imprecise because this could be extended to include greenhouses. Therefore, a more precise term may be (fully) “controlled environment growth systems” (CEGS).

Regardless of terminology, digital integration ensures control over individual parameters such as fertilizer composition and light (Huebbers and Buyel, 2021; Kaur et al., 2023) but requires sensors or even sensor networks that account for the discrete characters of individual plants (Huebbers and Buyel, 2021). For example, the metabolite and lipid composition of plants can be modulated by selecting specific light wavelengths for illumination (Rihan et al., 2022), and can thus help to reduce the concentration of potentially harmful metabolites like alkaloids (see Section 2.1.1). Furthermore, CEGS can be fully automated so that human intervention and ultimately the risk of contamination with human pathogens is minimized (Wirz et al., 2012; Huebbers and Buyel, 2021; Ren et al., 2023). The high degree of automation/mechanization in such systems, and the close proximity of the corresponding devices and plants, increases the likelihood that the product will come into contact with auxiliary and operating materials such as lubricants. Therefore, all devices should be designed to minimize such risks. This includes the selection of appropriate building materials, including steels compatible with food or pharmaceutical applications and plastics devoid of leachables (Jenke, 2002; Cuadros-Rodríguez et al., 2020; Zimmermann et al., 2021).

CEGS are overall the safest environment in which to produce biopharmaceutical proteins by PMF. The technology is scalable (e.g., several hundred kg of biomass can be processed per week (Holtz et al., 2015)), but the high investment and energy costs remain a significant bottleneck (Huebbers and Buyel, 2021; van Delden et al., 2021). This is especially relevant if the PMF products are not intended for pharmaceutical use, where the cost pressure on manufacturing is greater and low-investment infrastructure may be the only option to build economically viable processes.

### 3.4 Product extraction and purification

Although product purification is a key GMP requirement in PMF, at least for products that will be injected, purification also introduces some risks that must be mitigated during process design. A typical downstream processing sequence in PMF starts with harvesting and optional conditioning (e.g., washing (Ma et al., 2015) or blanching (Buyel et al., 2014a); Figure 1B). This is followed by initial extraction, which may involve further conditioning steps such as pH adjustment or flocculation (Buyel and Fischer, 2014b; Buyel and Fischer, 2014c; Buyel and Fischer, 2014d). The next major operation is clarification, which typically involves multiple filtration steps (Buyel and Fischer, 2014e), leading to product purification by two-phase extraction (Platis et al., 2008), membrane separation (Opdensteinen et al., 2018), chromatography (Buyel et al., 2012), or combinations thereof. The overall risk is that the sequence of downstream unit operations does not achieve the necessary purity due to the insufficient removal of process-related and/or product-related impurities, but each downstream operation poses specific risks to product safety that should be monitored and minimized during process development.

#### 3.4.1 Harvesting and conditioning

Manual harvesting processes carry an inherent safety risk because human operators come into close contact with the plant biomass containing the pharmaceutical product and may transfer pathogens. Therefore, personal protective equipment (in this case from the perspective of protecting the harvested biomass and product) should be worn at all times, including coats, gloves, hair nets and masks. In addition, the health of the operators should be monitored and staff should be encouraged to report any signs of illness to allow replacement and/or rescheduling. Although these are common routines for GMP-compliant processes based on cell cultures, they are also important in the context of intact plants because International Conference on Harmonisation (ICH) guidelines such as Q7^1^ (Good Manufacturing Practice Guidance for Active Pharmaceutical Ingredients) apply only to steps after plant harvesting and initial extraction. Even though plants do not support the replication of human viruses as discussed above, the harvesting of intact plants and parts such as leaves should ideally be fully automated as implemented in several CEGS, specifically indoor vertical farming (Wirz et al., 2012), because this minimizes operator-based contamination risks.

In some processes, the harvested biomass undergoes a thermal pretreatment described as blanching, in which the plant biomass is submerged in a hot (50°C–90°C) (Buyel et al., 2014a), potentially slightly acidic buffer (Opdensteinen et al., 2020), which will remove 50%–95% of tobacco host cell proteins (Buyel et al., 2016). This is an asset in terms of product safety but risks include partial or complete irreversible product denaturation accompanied by altered activity. An ill-designed blanching step may even increase protease activity and product degradation (Menzel et al., 2016; Menzel et al., 2018). Such conditioning steps should therefore be designed and implemented carefully, including the cross-checking of product activity in suitable assays.

#### 3.4.2 Extraction and further conditioning

Manual harvesting is usually accompanied by the manual transfer of biomass to the extraction device, so the protective measures discussed above should be maintained for this subsequent step. Biopharmaceuticals are usually extracted from plants and plant cells by homogenizing the biomass in the presence of a buffer (Buyel et al., 2015a). The latter controls pH and redox conditions to stabilize the product and to prevent unwanted modification by oxidation or reaction with plant-derived pigments and phenolic compounds. Oxidation and other unwanted reactions can also be suppressed by extraction in a nitrogen atmosphere (Ma et al., 2015). Protease inhibitors can be added during small-scale extraction (Menzel et al., 2016) or co-expressed in the plant cells (which also increases product accumulation (Jutras et al., 2016; Grosse-Holz et al., 2018)), but product integrity can be maintained simply by ensuring that all buffers are cooled to ∼10°C (Ma et al., 2015). Buffer cooling is beneficial because nothing is added to the process, but additional equipment will be required for this step. In contrast, any inhibitors are additional contaminants that need to be removed later. The same holds true for extraction techniques that do not require buffers at all, such as the use of a screw press (Buyel and Fischer, 2014d). However, the conditions in the resulting green juice can negatively affect the product and its activity, for example, due to the low pH (e.g., ∼5.5 in case of tobacco). As for blanching, the implementation of such methods should therefore be accompanied by a careful assessment of the impact on product stability and activity.

An extract can then be conditioned to facilitate subsequent clarification and purification by pH adjustment as well as the addition of flocculants and/or filter aids. The latter are often large, inert cellulose fibers that are easily removed during subsequent purification steps (Buyel et al., 2014b), whereas flocculants are highly charged polymers (Buyel and Fischer, 2014c) that can bind to proteins (Jurjevec et al., 2023) and escape detection. The highest purity grades should therefore be used with pharmaceutical products to ensure product safety. Although non-pharmaceutical products like food additives also need to comply with good manufacturing practices (Manning, 2018), the purity and safety requirements are usually less stringent, and this should be taken into account when selecting the reagents in order to align raw material quality and safety requirements.

#### 3.4.3 Clarification and purification

Clarification (mostly filtration) and purification steps typically remove particles, including viruses, as well as soluble host cell components such as proteins. Therefore, both operations inherently increase the safety of biopharmaceuticals. Nevertheless, processes based on bacteria or animal cell hosts have revealed that both steps also introduce risks in terms of product safety. For example, equipment can release leachables and extractables and should be selected to minimize these risks, taking into account the properties of plant extracts such as the presence of phenolic compounds. Specifically, protein A, the common affinity ligand for antibody capture, can be found as a process-related impurity in antibody elution fractions (Carter-Franklin et al., 2007) and exposure to phenolics can result in the permanent discoloration of chromatography resins.

Another major risk to product safety is presented by the hold times that are required during extract processing. For example, even many continuous processes use intermediate storage tanks that allow time buffering between individual downstream steps (such as two in-series filters) to compensate for fluctuations in volumetric fluxes. The flow regime in such tanks is far from an ideal plug flow and will create a broad residence time distribution (Sencar et al., 2020; Lali et al., 2022). Therefore, some of the product will be held substantially longer in the process than might be expected based on the average residence time. This is critical during the early purification stages, when host cell proteases or oxidases are still abundant and can act on and modify the product, potentially compromising its activity and safety. Cooling the process intermediates can reduce such unwanted enzyme activities but this requires additional equipment. A fully continuous process without hold tanks requires sophisticated process control, and this is vulnerable to errors that create new product safety risks. Comprehensive risk management is therefore necessary during process development (Sparrow et al., 2013; Zalai et al., 2013; Qiu et al., 2015; Luo et al., 2021).

## 4 Safety-relevant product properties

The target product profile of biopharmaceuticals produced by PMF is based on the same aspects stipulated for other bioprocesses, such as efficacy and safety^2^. Both depend on the molecular properties of the product, such as sequence integrity, folding, and PTMs such as disulfide bonds, phosphorylation and glycosylation. The latter can affect folding, but in the context of product safety the major concern is immunogenicity because plant *N*-linked glycans differ from those found in mammals in several fundamental ways, including the presence of xylose residues (not found in mammals) and the linkage of fucose via an α3 glycosidic bond (an α6 bond is found in mammals) (Strasser, 2016). Furthermore, *O*-linked glycans in plants are mostly found on hydroxyproline residues whereas serine and threonine are the preferential targets in mammals. These non-native glycans can trigger immune responses when recombinant human proteins produced by PMF are injected (Bardor et al., 2003; Jin et al., 2008). However, there is no evidence that the elicitation of anti-glycan antibodies is harmful (Shaaltiel and Tekoah, 2016; Rup et al., 2017). The situation may be different in patients with a history of allergy (Schwestka et al., 2021), who may be especially sensitive to plant-derived glycan structures. Similarly, persons with allergies against egg proteins, including glycoproteins such as ovotransferrin and ovomucoid (Hwang et al., 2014), may exhibit mild but unwanted allergy-like side effects (not anaphylaxis) when receiving influenza vaccines produced in eggs (James et al., 1998; Gruenberg and Shaker, 2011). To address this, various plant (cell) lines have been developed that lack plant-specific glycosyltransferases (Strasser et al., 2004; Jansing et al., 2018) and in some cases also incorporate human enzymes to make the glycans not only human-compatible but fully humanized (Castilho et al., 2013; Montero-Morales and Steinkellner, 2018). The corresponding PMF products may achieve greater activity, as reported for at least one vaccine candidate (Pantazica et al., 2023).

## 5 Breeding and genetic engineering targets to address safety aspects

Breeding and genetic engineering can be used to reduce several of the safety risks discussed above, and not only by the modification of glycans. Specifically, plant proteases can be inactivated to enhance product integrity (e.g., to minimize degradation and aggregation) or they can be expressed in a targeted manner to ensure precise processing, such as the removal of leader sequences, as demonstrated for transforming growth factor β1 (Goulet et al., 2012; Wilbers et al., 2016). Similarly, enzyme cascades synthesizing toxic compounds can be interrupted, as demonstrated by the creation of nicotine-free tobacco (Schachtsiek and Stehle, 2019). One can also learn from other host systems and knock out host cell proteins that are difficult to remove during downstream processing (Chiu et al., 2017), including but not limited to those that are toxic or allergenic as discussed above. These and other options such as the use of chaperones to promote correct protein folding or modifications to prevent oxidation, all of which improve the performance of host species in terms of product yield, activity and safety, have been reviewed in detail elsewhere (Buyel et al., 2021; Singh et al., 2021). The CRISPR/Cas9 system and its regulatory implications have been thoroughly assessed in the context of PMF (Bortesi and Fischer, 2015; Eckerstorfer et al., 2019; Fiaz et al., 2021).

Importantly, such safety-improving genetic engineering steps must be balanced against, for example, the viability and productivity of the resulting plant (cell) line. For example, it is desirable to knock out proteases as discussed above because they can trigger the (partial) degradation of a PMF product, thus reducing its activity (Donini et al., 2015; Mandal et al., 2016; Menzel et al., 2018). However, proteases fulfil essential biological functions and may be required for germination and plant growth (Martinez et al., 2019; van der Hoorn and Klemenčič, 2021), which are important to achieve a high product yield. Therefore, process engineering rather than genetic engineering may be more suitable in some instances.

Furthermore, single knockouts may not be sufficient due to redundancies in metabolic pathways. For example, the morphine biosynthesis pathway branches when it reaches the intermediate thebaine, which may be converted to morphine via codeinone or morphinone (Ziegler et al., 2009; Onoyovwe et al., 2013). Therefore, at least one enzyme in each branch must be knocked out to block morphine synthesis completely. In this context, inactivating a certain enzyme cascade may result in a re-direction of the metabolic flux to other metabolites that can be toxic too.

## 6 Conclusion and outlook

Plants, like all other biological hosts, present certain product safety risks due to their natural components (e.g., toxic metabolites), cultivation conditions, and differences in PTMs. It is important to monitor these risks when operating GMP-compliant manufacturing processes and to implement a suitable risk management strategy. Such a strategy should focus on identifying and prioritizing risks based on the specifics of a given process, e.g., protein (Bracewell et al., 2015) or low-molecular-mass impurities (Luo et al., 2021). Because prioritization will depend on product properties and the characteristics of the manufacturing process, it is not useful to provide general recommendations other than the established concepts and heuristics such as failure mode effects (and criticality) analysis (FME(C)A) or hazard analysis and critical control points (HACCP) as outlined in the ICH Q9 guidelines^3^. However, such a risk assessment could benefit greatly from structured and curated databases that aggregate, for example, information on phytotoxins (Günthardt et al., 2018), because knowledge and ultimately product safety will increase as more and more processes are developed. Also, general knowledge about such impurities and contaminants (i.e., excluding specific process steps or conditions) should have a pre-competitive character and should thus be disclosable by the companies involved. Financing the curation and maintenance of such a database is more likely to be a bottleneck.

When looking at the individual safety aspects discussed in Sections 2–5, none of the risks is grave enough to prevent the use of plants for PMF applications. Indeed, such risks are easily mitigated by implementing established risk management and process design principles. Overall, plants can be regarded as safe host systems for PMF, and the selection of food or feed crops can exclude many of the risks associated with hosts that produce intrinsic toxic components.

## Footnotes

1
https://www.ema.europa.eu/en/documents/scientific-guideline/ich-q-7-good-manufacturing-practice-active-pharmaceutical-ingredients-step-5_en.pdf

2
https://www.who.int/observatories/global-observatory-on-health-research-and-development/analyses-and-syntheses/target-product-profile/links-to-who-tpps-and-ppcs

3
https://www.ema.europa.eu/en/documents/scientific-guideline/international-conference-harmonisation-technical-requirements-registration-pharmaceuticals-human-use_en-3.pdf

## Abbreviations

CEGS, controlled environment growth systems; CHO, Chinese hamster ovary; HCP, host cell protein; PMF, plant molecular farming; PTM, post-translational modification.
